# High total volatile organic compounds pollution in a hospital dental department

**DOI:** 10.1007/s10661-017-6265-z

**Published:** 2017-10-18

**Authors:** Ming-Hui Liu, Tao-Hsin Tung, Fen-Fang Chung, Li-Chuan Chuang, Gwo-Hwa Wan

**Affiliations:** 1 Department of Pediatric Dentistry, Taoyuan Chang Gung Memorial Hospital, Taoyuan, Taiwan Republic of China; 2 Department of Medical Research and Education, Cheng-Hsin General Hospital, Taipei, Taiwan Republic of China; 3 Department of Nursing, Chang Gung University of Science and Technology, Taoyuan, Taiwan Republic of China; 4 Department of Pediatric Dentistry, Linkuo Chang Gung Memorial Hospital, Taoyuan, Taiwan Republic of China; 5 Department of Respiratory Therapy, College of Medicine, Chang Gung University, Taoyuan, Taiwan Republic of China; 6 Department of Respiratory Therapy, Chang Gung University of Science and Technology, Chiayi, Taiwan Republic of China; 7 Department of Neurosurgery, Linkou Chang Gung Memorial Hospital, Taoyuan, Taiwan Republic of China

**Keywords:** Indoor air quality, Dental department, Particulate matter, Volatile organic compounds, Bacteria

## Abstract

Bioaerosols produced by dental procedures may affect indoor air quality and cause infections in dental healthcare workers. To provide air quality data that can be used to protect dental healthcare workers, this study evaluated the air quality and its influencing factors in the dental department of the Chang Gung Memorial Hospital in Taiwan. The study was a cross-sectional study design. Indoor air quality (IAQ) evaluations were conducted in six locations: pediatric dentistry, craniofacial orthodontic dentistry, periodontal dentistry, and general practice dentistry, instrument washing room, and patient waiting area. The measured air quality parameters included temperature, relative humidity, and concentrations of CO_2_, total volatile organic compounds (TVOCs), suspended particulate matter (PM), and bacteria. TVOCs concentrations at all six sampling stations were found to exceed the indoor air quality standards prescribed by the Taiwan Environmental Protection Agency. The highest concentrations of atmospheric PM_10_, PM_2.5_, and PM_1_ were found in the periodontal dentistry department, while the lowest concentrations occurred in the patient waiting area. The detection rate for Gram-positive bacteria was highest in the pediatric department (25%) and lowest in the instrument washing room (9%). *Micrococcus luteus* and *Bacillus cereus* were the primary pathogens detected. The dental departments of the hospital had a serious TVOCs pollution. The air quality of dental departments deserves long-term surveillance and attention.

## Introduction

Dental treatments include many diverse procedures, such as restorative dentistry, root canal therapy, ultrasonic scaling, periodontal curettage of dental calculus, periodontal surgery, prosthetic dentistry, orthodontic treatment, surgical extraction of impacted third molars, and dental implant surgery. The use of high-speed drills or ultrasonic scalers can produce aerosols. Microbial aerosols and splatters are also generated during dental procedures. These aerosols are air-suspended liquid or solid molecules that contain bacteria, viruses, fungi, saliva, and blood. Aerosols produced during dental procedures not only reduce the IAQ, but also pose a threat to the health of dental staff and are important sources of infection (Szymańska 2007; Bennett et al. 2000; Leggat et al. 2007).

IAQ assessment indicators include concentrations of CO_2_ (Li et al. 2001; Scheff et al. 2000), PM, TVOCs, bacteria (Liu et al. 2000), fungi, and viruses, as well as temperature and relative humidity (RH). Particle concentrations in the indoor air of a hospital are related to human activity, air exchange, and air filtration (Streifel et al. 1989). High concentrations of aerosols can be produced by a high-speed drill or rotating instrument used inside an oral cavity (Harrel and Molinari 2004; Kedjarune et al. 2000; Leggat and Kedjarune 2001). The composition, concentration, and distribution of aerosols is affected by factors such as the type of treatment procedure, size and location of the treatment room, duration of treatment, mode of treatment, patient characteristics, and seasonality (Bennett et al. 2000; Kedjarune et al. 2000; Grenier 1995).

In one study, closed and isolation dental clinic rooms had high bacterial concentrations ((216 ± 75) CFU m^−3^ during scaling and (75 ± 22) CFU m^−3^ during fillings). The bacterial concentration after 2 h of dental treatment in the isolation dental treatment room was the same as the background concentration of 12–14 CFU m^−3^ (Grenier 1995). In the same study, bacteria were detectable in all areas of an open clinic with multiple dental chairs. Peak bacterial concentrations were observed in the main treatment area after 3 h of dental treatment (76–114 CFU m^−3^), and the amount of bacteria in the non-treatment area (42 CFU m^−3^) remained higher than the background level. This shows that aerosols can spread and move through the air (Grenier 1995). All dental procedures, and particularly dental surgery, are intended to be performed in a sterile environment. The use of protective measures, such as sterile gloves, gowns, and face masks, is very important during these procedures.

The climate in Taiwan is characterized by high temperature and humidity, which are very conducive to the formation of bioaerosols. Patients, family members, and healthcare workers are the main sources of biological aerosols in hospitals. The close contact between patients and medical staff, coupled with the central air-conditioning systems typically used in hospitals, increases the probability of mutual infection. To date, few studies have evaluated the IAQ of dental departments in hospitals. The purpose of this study was to investigate the air quality parameters and aerosol distribution in six different locations of the dental department: pediatric dentistry, craniofacial orthodontic dentistry, periodontal dentistry, general practice dentistry, instrument washing room, and patient waiting area.

## Materials and methods

### Sampling locations

Permits for this study were obtained from the Taoyuan Chang Gung Memorial Hospital in northern Taiwan. This study evaluated the IAQ for the six locations of the dental department, including pediatric dentistry (PEDI), craniofacial orthodontic dentistry (ORTHO), periodontal dentistry (PERIO), general practice dentistry (GP), the instrument washing room (IR), and the patient waiting area (PWA).

**Fig. 1:**
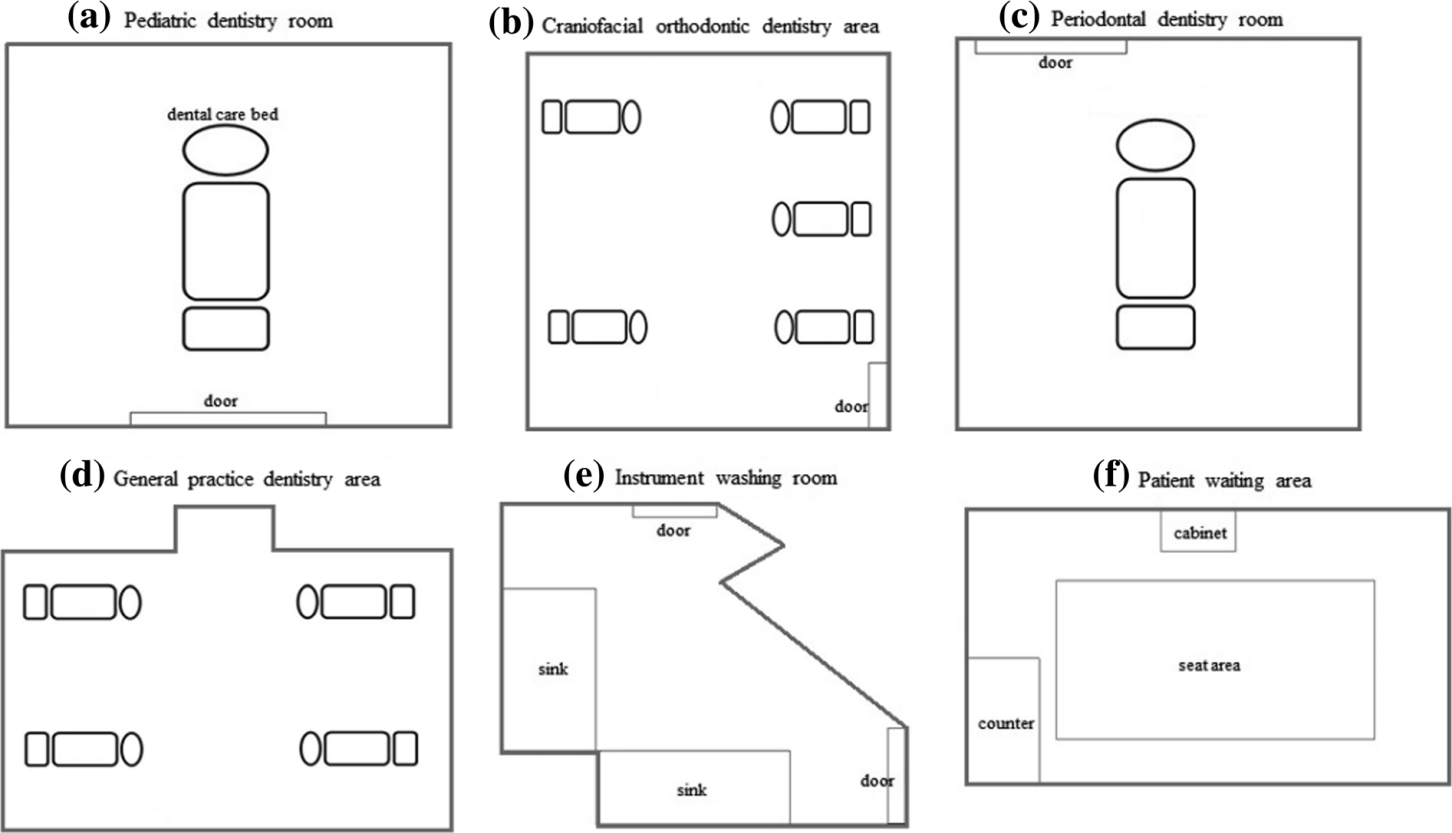
Diagram of the six locations of the dental department

Figure 1 shows the six dental departments of the selected hospital. The space volumes of PEDI, PERIO, IR, ORTHO, GP, and PWA were 26.51, 39.22, 31.05, 96.59, 232.03, and 78.62 m^3^, respectively. During the sampling period, indoor air was conditioned but not heated.

### Air quality monitoring

This study was performed from July to August in 2016. Indoor air quality parameters of six locations in the dental department were monitored for 9 h (8 am to 5 pm) per day for 3 days. Bacterial samples were collected twice a day (in the morning and in the afternoon) for 3 days. The air sampling instruments were placed approximately 1.5 m from the dental unit to avoid interrupting dental treatment, and as close to the center of the sampling area as possible. All instruments were positioned 1 m above the floor to simulate the seated breathing zone of healthcare workers.

The evaluated air quality indices included the air temperature, RH, and concentrations of TVOCs, CO_2_, suspended PM, and bacteria. The air temperature, RH, and CO_2_ concentration were determined every minute using a digital psychrometer (TSI, Inc., Shoreview, MN, USA). The PM levels were measured every 6 s using a portable dust monitor with 31 size channels measured the size range between 0.25 and 32 μm (Model 1.110; Grimm Labortechnik Ltd., Ainring, Germany). The level of TVOCs was determined every minute by a hand carried detector (ppb RAE 30000, USA). Bacterial concentrations were assessed using Andersen one-stage viable impactors (N6; Andersen Samplers, Atlanta, Georgia) with 20 mL of tryptic soy agar at an airflow rate of 28.3 L min^−1^ for 3 min. Duplicate bacterial samples were collected to ensure sampling accuracy, and bacterial samples were incubated at 30 ± 1 °C for 48 ± 2 h, as recommended by the Taiwan Environmental Protection Agency (TEPA). The positive hole conversion table and sampled air volume were used to calculate the number of colony forming units per volume of air (CFU m^−3^). All the bacterial colonies were identified biochemically.

### Statistical analysis

Data were analyzed using SPSS version 21.0 (IBM Corp., Armonk, NY, USA). All figures were constructed with GraphPad Prism version 6.0 (GraphPad Software, San Diego, CA, USA). The two-sided *p* value with *α* < 0.05 was considered statistically significant. Besides the bacterial concentration, the hourly data of air quality indices were used for statistical analysis in the study. The PM was classified as PM_10_ (aerodynamic diameter ≤ 10 μm), PM_2.5_ (aerodynamic diameter ≤ 2.5 μm), and PM_1_ (aerodynamic diameter ≤ 1 μm). The IAQ indices in the six locations of the dental department in the hospital were compared using the one-way analysis of variance test with Scheffe’s post hoc comparison or Kruskal-Wallis test for continuous variables. Pearson correlation analysis was applied to identify the relationship between combinations of two continuous variables with normally distributed data.

## Results

During the study period, the largest and smallest numbers of people were observed in ORTHO (*N* = 105) and in PERIO (*N* = 66), respectively. Air quality data collected from the six locations of the dental department (*n* = 156) is shown in Table 1. The highest temperature was recorded in PEDI (24.5 °C), while the lowest was recorded in ORTHO (20.43 °C). The temperature differences in the six locations are statistically significant (*p* < 0.001). The IR had the highest RH value (70.59%), while PERIO had the lowest RH value (58.11%). Differences in the RH values in the six locations were statistically significant (*p* < 0.001). The measured CO_2_ concentrations in six regions ranged from 491.73 to 653.65 ppm, with the maximum occurring in PERIO and the minimum occurring in IR. A significant difference (*p* < 0.001) was found in the CO_2_ concentrations for the six locations, but all values were within the TEPA indoor air quality standard of 1000 ppm.

**Table 1:** Distribution of air quality indices in the six dental departments of Chang Gung Memorial Hospital

Variables	PEDI (*n* = 25)	ORTHO (*n* = 27)	PERIO (*n* = 23)	GP (*n* = 27)	IR (*n* = 27)	PWA (*n* = 27)	*p* value
Temperature, °C	24.5 (0.20)	20.43 (2.23)*	22.66 (0.71)*^†^	21.42 (1.07)*^‡^	22.06 (0.63)*^†^	24.28 (0.23)^†‡§¶^	< 0.001
RH, %	64.89 (2.89)	67.15 (2.27)*	58.11 (1.29)*^†^	67.90 (2.16)*^‡^	70.59 (0.78)*^†‡§^	60.24 (1.82)*^†‡§¶^	< 0.001
CO_2_, ppm	521.74 (44.03)	523.71 (43.43)	653.65 (90.57)*^†^	601.20 (82.67)*^†^	491.73 (84.63) ^‡§^	630.80 (86.73)*^†¶^	< 0.001
TVOCs, ppb	930.09 (277.67)	1083.97 (665.40)	1329.03 (439.00)	1373.99 (450.79)*	674.56 (191.20)^†‡§^	947.11 (228.92)^§^	< 0.001
PM_10_, μg m^−3^	18.02 (3.41)	13.21 (5.45)	28.12 (4.47)*^†^	21.66 (9.63)^†‡^	25.65 (9.21) *^†^	14.70 (2.67)^‡§¶^	< 0.001
PM_2.5_, μg m^−3^	13.27 (2.28)	11.14 (4.44)	19.82 (4.25)*^†^	17.97 (6.37)*^†^	15.53 (5.19)^†‡^	8.87 (1.45)*^‡§¶^	< 0.001
PM_1_, μg m^−3^	12.05 (2.44)	10.24 (4.04)	17.78 (4.74)*^†^	17.20 (6.00)*^†^	12.98 (4.67)^‡§^	7.80 (1.41)*^‡§¶^	< 0.001
Bacteria, μg m^−3#^	247.97 (88.36–262.00)	84.48 (48.42–236.81)	773.01*^†^ (405.26–1348.84)	307.56*^†^ (259.94–1524.85)	619.63*^†^ (471.36–855.61)	1299.25*^†¶^ (952.25–1697.97)	*< 0.001*

The data were presented as mean (SD) or median (25–75 percentiles)

*PEDI* pediatric dentistry, *ORTHO* craniofacial orthodontic dentistry, *PERIO* periodontal dentistry, *GP* general practice dentistry, *IR* instrument washing room, *PWA* patient waiting area

*compared to PEDI, ^†^compared to ORTHO, ^‡^compared to PERIO, ^§^compared to GP, ^¶^compared to IR, *p* ≤ 0.05. ^#^the sample size for each location was 6

The average TVOCs concentrations found in the six sampling locations all exceeded TEPA indoor air quality standards of 560 ppb h^−1^. The highest concentration occurred in GP (1373.99 ppb) and the lowest occurred in the IR (674.56 ppb). The TVOC concentrations in the six locations were significantly different (*p* < 0.001). The maximum concentrations of PM_10_, PM_2.5_, and PM_1_ were found in PERIO, while the minimum concentrations were found in the PWA. Concentrations of PM_10_, PM_2.5_, and PM_1_ had statistically significant differences between the six sampling locations (*p* < 0.001). However, none of the values exceeded the TEPA standard. The upper limit for the PM_10_ concentration was 75 μg m^−3^ for 24-h average concentration, while that for PM_2.5_ was 35 μg m^−3^ for 24-h average concentration.

The median concentrations of airborne bacteria in PERIO (773.01 CFU m^−3^), GP (307.56 CFU m^−3^), IR (619.63 CFU m^−3^), and PWA (1299.25 CFU m^−3^) were significantly higher than those in ORTHO (84.48 CFU m^−3^) and PEDI (247.97 CFU m^−3^). Also, a significant difference in the airborne bacterial concentration was found between PWA and IR (*p* = 0.025). In this study, the bacterial concentrations were generally lower than the TEPA indoor air quality standards, which sets an upper limit of 1500 CFU m^−3^. Only two specimens exceeded the 1500 CFU m^−3^ standard, both during afternoon sessions, one in GP (4058.67 CFU m^−3^) and one in the PWA (2551.39 CFU m^−3^) (data not shown). In the morning and afternoon sessions in PEDI and ORTHO, bacterial concentrations were below 500 CFU m^−3^.

Temperature had a significant negative correlation with RH (*r* = − 0.463, *p* < 0.01) and TVOCs (*r* = − 0.16, *p* < 0.05), while RH had a significant negative correlation with CO_2_ (*r* = − 0.550, *p* < 0.01) and TVOC (*r* = − 0.172, *p* < 0.05) concentrations (Table 2). In addition, CO_2_ concentration was positively correlated with the concentration of TVOCs (*r* = 0.377, *p* < 0.01), PM_10_ (*r* = 0.28, *p* < 0.01), and PM_2.5_ (*r* = 0.16, *p* < 0.05). PM_10_ had significant positive correlations with PM_2.5_ (*r* = 0. 827, *p* < 0.01) and PM_1_ (*r* = 0. 739, *p* < 0.01).

**Table 2:** Associations of air quality indices in the six dental departments

	(1)	(2)	(3)	(4)	(5)	(6)	(7)	(8)	(9)
(1) Temperature	1								
(2) RH	− 0.463**	1							
(3) CO_2_	− 0.008	− 0.550**	1						
(4) TVOCs	− 0.160*	− 0.172*	0.377**	1					
(5) PM_10_	0.013	− 0.056	0.280**	0.115	1				
(6) PM_2.5_	− 0.110	0.031	0.160*	0.131	0.827**	1			
(7) PM_1_	− 0.126	0.043	0.139	0.140	0.739**	0.985**	1		
(8) Bacteria	0.194	− 0.147	0.139	0.058	0.122	0.113	0.118	1	
(9) Number of people	0.087	0.062	0.006	− 0.073	− 0.226	− 0.360	− 0.378*	− 0.177	1

*p < 0.05, **p < 0.01

The types and isolation rates of bacteria in the six dental departments are listed in Table 3. The total airborne bacteria species include both Gram-positive and Gram-negative bacteria. Most of the bacteria were Gram-positive species (94%). The proportion of Gram-positive bacteria was largest in PEDI (25%) and smallest in the IR (9%). The Gram-positive bacteria included *Micrococcus luteus* (31%), *Bacillus cereus* (22%), *Bacillus circulans* (9%), other *Bacillus* spp. (6%), *Micrococcus lylae* (6%), other *Micrococcus* spp. (6%), *Achromobacter* spp. (3%), *Bacillus licheniformis* (3%), *Staphylococcus haemolyticus* (3%), and *Staphylococcus kloosii* (3%). Gram-negative bacteria, which included *Brevundimonas diminuta* and *Nocardia* spp., were only found in GP and represented a mere 6% of the population.

**Table 3:** Isolation rates of airborne bacteria (%) in the six dental departments

Airborne bacterial species	Isolation rate^✝^						
	Total	PEDI	ORTHO	PERIO	GP	IR	PWA
Gram-positive bacteria	30/32 (94%)	8 (25%)	6 (19%)	4 (13%)	5 (16%)	3 (9%)	4 (13%)
*Achromobacter* species	1/32 (3%)	0 (0%)	1 (3%)	0 (0%)	0 (0%)	0 (0%)	0 (0%)
*Bacillus cereus*	7/32 (22%)	5 (16%)	1 (3%)	0 (0%)	0 (0%)	1 (3%)	0 (0%)
*Bacillus circulans*	3/32 (9%)	1 (3%)	0 (0%)	0 (0%)	1 (3%)	0 (0%)	1 (3%)
*Bacillus licheniformis*	1/32 (3%)	0 (0%)	0 (0%)	1 (3%)	0 (0%)	0 (0%)	0 (0%)
*Bacillus* species	2/32 (6%)	0 (0%)	1 (3%)	1 (3%)	0 (0%)	0 (0%)	0 (0%)
*Micrococcus luteus*	10/32 (31%)	2 (6%)	2 (6%)	1 (3%)	2 (6%)	1 (3%)	2 (6%)
*Micrococcus lylae*	2/32 (6%)	0 (0%)	0 (0%)	1 (3%)	1 (3%)	0 (0%)	0 (0%)
*Micrococcus* species	2/32 (6%)	0 (0%)	0 (0%)	0 (0%)	0 (0%)	1 (3%)	1 (3%)
*Staphylococcus haemolyticus*	1/32 (3%)	0 (0%)	0 (0%)	0 (0%)	1 (3%)	0 (0%)	0 (0%)
*Staphylococcus kloosii*	1/32 (3%)	0 (0%)	1 (3%)	0 (0%)	0 (0%)	0 (0%)	0 (0%)
Gram-negative bacteria	2/32 (6%)	0 (0%)	0 (0%)	0 (0%)	2 (6%)	0 (0%)	0 (0%)
*Brevundimonas diminuta*	1/32 (3%)	0 (0%)	0 (0%)	0 (0%)	1 (3%)	0 (0%)	0 (0%)
*Nocardia* spp*.*	1/32 (3%)	0 (0%)	0 (0%)	0 (0%)	1 (3%)	0 (0%)	0 (0%)

*PEDI* pediatric dentistry, *ORTHO* craniofacial orthodontic dentistry, *PERIO* periodontal dentistry, *GP* general practice dentistry, *IR* instrument washing room, *PWR* patient waiting area

^✝^The number of specific isolated microorganism divided by the total number of isolated microorganisms

## Discussion

Hospital indoor air pollution is associated with inadequate building environments, including building materials, air-conditioning systems, and ventilation rates, and with human factors, such as overcrowding in constrained spaces. This was the first study in Taiwan evaluating air quality indices for a dental department in a hospital.

Dentists face potential exposure to various air pollutants during different types of dental procedures. Studies have shown that dental workers have a higher chance of exposure to microbes and, consequently, a higher risk of respiratory infections, inflammation, and disease than do other workers (Bennett et al. 2000). In addition, skin irritation and eye infections are often a health hazard for dental staff (Leggat et al. 2007). The distance between the dentist and the patient, the location of the patient, and the patient head height during dental treatment also affected the distribution and concentration of aerosols. Studies have shown that the central location of a dentist’s face (including the eyes and nasal region) are at high-risk for infection (Nejatidanesh et al. 2013). Dental rubber barriers used in mouth during patient treatment can effectively reduce aerosol concentrations (Pina-Vaz et al. 2008; Al-Amad et al. 2016; Tag and El-Hady 1997), while patients using antibacterial mouthwash before dental treatment also proved to decrease aerosolized bacterial production effectively (Fine et al. 1992; Molinari and Molinari 1992; Fine et al. 1993).

In our study, the TVOCs concentrations of the six sampling locations exceeded the TEPA indoor air quality standard, and the highest TVOCs concentration was found in GP. Resin materials are often used for temporary prosthetics and relining removable dentures in this department. One of the main components of resin monomer is methyl methacrylate, which has a strong acrid smell and volatile character. The resin monomer spreads through the air following polymerization and incomplete setting. This monomer is the main source of volatile organic compounds in GP, which has an open area floor plan with five dentists working simultaneously. The partial pressure of resin monomer vapor is thus very high from the volatile gas accumulation in this location.

The second highest value of TVOCs was recorded in PERIO, which is a closed treatment room. Common treatments here include ultrasonic scaling, periodontal surgery, and dental implant surgery, none of which include volatile materials. However, 3 days a week, there are four prosthetic dentists who treat patients in this department. They often use resin monomers to make temporary prosthetics and adjust dentures. The volatile gas therefore spreads through the air-conditioning system. In addition, every Wednesday afternoon, the nurses use bleaching solvent to disinfect the boxes used for collecting surgery devices, and they throw away the bleach the following day. We speculate that the resin monomer and the bleaching solvent both contributed to PERIO having the second highest concentration of TVOCs. In ORTHO, the dentists also use resin materials to reline newborn cleft palates. In addition, orthodontic and pediatric dentists often use resin-based adhesive materials and bonding agents, which have volatile properties. The lowest concentration of TVOCs occurred in the IR because only enzyme powder is used to soak and clean all instruments, so no volatile material sources were found in this location.

In order to prevent the formation of TVOCs in dental departments, high-power ventilation can be used along with a high-density charcoal filter system near the dentistry procedure locations. A method for preventing high TVOC concentrations during dental treatment is still needed. One suggestion is that all dentists should close the button of resin monomer immediately after using it. The correct powder to liquid ratio would also help prevent unnecessary emissions. High values of TVOCs are harmful to the working staff and patients. Hospitals need to focus more on improving the air quality in their dental departments. Additionally, CO_2_ levels are closely related to TVOC, PM_10_, and PM_2.5_ concentrations. With a better ventilation system, the CO_2_ level is decreased, and the volatile solvents and aerosols would be more effectively flushed and filtered out.

Dental scaling can produce high concentrations of aerosols, which can be removed using high-intensity evacuators (Barnes et al. 1998; Harrel et al. 1996; Harrel et al. 1998). High-intensity suction is thus a good way to reduce the risk of exposure to microbes. Bioaerosols collected in the dental locations included *Streptococcus mutans* and *Streptococcus sanguinis* bacteria, mainly from the patient mouths (Earnest and Loesche 1991), as well as fungi from the environment (Krogulski and Szczotko 2010). Adding a fluorescent agent to the water column of a high-speed drill would indicate when splashing has occurred on the head, upper arm, neck, or chest of the dentist, which is a problem especially when treating the lower right molar area. Most of the sputtering landed on the patient chest (Bentley et al. 1994). In this study, we found bacterial counts as high as 4000 CFU m^−3^ in GP one afternoon. Because the number of patients that day was up to 20 people simultaneously, the airborne bacterial level was particularly high. For each afternoon session in the PWA, a higher bacterial concentration was noted, possibly because more patients and families were present. During afternoon sessions in PEDI and ORTHO, bacterial concentrations were all below 500 CFU m^−3^. We infer that this was because of a lower number of patients on weekdays. There were many airborne bacterial species in PEDI and ORTHO, due to the larger number of people and children present.

In conclusion, serious TVOCs pollution was found in the dental departments of the hospital. The air quality of dental departments deserves attention and requires long-term surveillance from environmental safety and health departments in hospitals to protect patients as well as dentists and other nursing workers. The distributions of air pollutant concentrations during common dental treatment procedures should be evaluated in a future study.

## Abbreviations

TVOCs: total volatile organic compounds
PM: suspended particulate matter
RH: relative humidity
PEDI: pediatric dentistry
ORTHO: craniofacial orthodontic dentistry
PERIO: periodontal dentistry
GP: general practice dentistry
IR: instrument washing room
PWA: patient waiting area
TEPA: Taiwan Environmental Protection Agency
